# Mountain pine beetle selectivity in old-growth ponderosa pine forests, Montana, USA

**DOI:** 10.1002/ece3.522

**Published:** 2013-03-13

**Authors:** Paul A Knapp, Peter T Soulé, Justin T Maxwell

**Affiliations:** 1 Carolina Tree-Ring Science Laboratory, Department of Geography, University of North Carolina GreensboroGreensboro, 27401, North Carolina; 2 Department of Geography and Planning, Appalachian State University, Tree-Ring Science LaboratoryBoone, North Carolina, 28608; 3 Department of Geography, Indiana UniversityBloomington, Indiana, 47405

**Keywords:** Basal area increment, growth divergence, intrinsic water-use efficiency, Montana, mountain pine beetle outbreak, Ponderosa pine, USA

## Abstract

A historically unprecedented mountain pine beetle (MPB) outbreak affected western Montana during the past decade. We examined radial growth rates (AD 1860–2007/8) of co-occurring mature healthy and MPB-infected ponderosa pine trees collected at two sites (Cabin Gulch and Kitchen Gulch) in western Montana and: (1) compared basal area increment (BAI) values within populations and between sites; (2) used carbon isotope analysis to calculate intrinsic water-use efficiency (iWUE) at Cabin Gulch; and (3) compared climate-growth responses using a suite of monthly climatic variables. BAI values within populations and between sites were similar until the last 20–30 years, at which point the visually healthy populations had consistently higher BAI values (22–34%) than the MPB-infected trees. These results suggest that growth rates two–three decades prior to the current outbreak diverged between our selected populations, with the slower-growing trees being more vulnerable to beetle infestation. Both samples from Cabin Gulch experienced upward trends in iWUE, with significant regime shifts toward higher iWUE beginning in 1955–59 for the visually healthy trees and 1960–64 for the MPB-infected trees. Drought tolerance also varied between the two populations with the visually healthy trees having higher growth rates than MPB-infected trees prior to infection during a multi-decadal period of drying summertime conditions. Intrinsic water-use efficiency significantly increased for both populations during the past 150 years, but there were no significant differences between the visually healthy and MPB-infected chronologies.

## Introduction

The ongoing mountain pine beetle (MPB; *Dendroctonus ponderosae* Hopkins) epidemic affecting pine species in western North America is the largest in recorded history (Mitton and Ferrenberg 2012). During 2001–2010, over 2.3 million hectares of forests in Montana were affected by the MPB (Chaney 2011), which caused mortality to numerous species of the *Pinus* genus including lodgepole (*P. contorta*), ponderosa (*P. ponderosa*), and whitebark (*P. albacaulis*) pines (Logan et al. 2010). Mortality rates peaked in 2009 when approximately 1.5 million hectares of pines experienced MPB-caused death. The MPB is native to western North America, with occasional years marked by outbreak populations (Brunelle et al. 2008) triggered by favorable periodic climatic and/or stand conditions (Gibson et al. 2009), but typically only low levels of infestation and subsequent tree mortality occur in a given year.

The magnitude of the current epidemic has been attributed to multi-decadal climate change (Logan and Powell 2001; Akuma et al. 2008; Bentz et al. 2010) directly through a reduction in low temperature-induced mortality caused by extended cold-air incursions, particularly during the autumn and spring, and indirectly through increased drought frequency. Carbon assimilation is impacted by reduced stomatal conductance and reduced photosynthate allocations for defense mechanisms or tissue repair during drought periods, making the infected trees more susceptible to mortality. Additionally, warmer conditions have allowed a change from univoltine to bivoltine life cycles (one to two generations) in some broods of MPB (Mitton and Ferrenberg 2012). Extensive fire-suppression activities that decrease the number and extent of stand-replacing fires can also affect forest susceptibility to MPB outbreaks (Kulakowski et al. 2012).

During the summer months, MPB attack trees en-mass and girdle the phloem that in turn decreases photosynthate transport within a tree and reduces tree health (Gibson et al. 2009). MPB also carry the spores of two “blue stain” fungal species, *Ophiostoma montium* and *Grosmannia clavigera*, that are inoculated into the trees by adult beetles. The fungi subsequently spread in the sapwood and phloem of infected trees, reducing water transport and altering resin flow in a fashion that contributes to an increase in tree mortality (Gibson et al. 2009). Mass-attacked trees typically die the following growing season, although trees subject to partial or “strip” attacks may survive several years post infestation (Gibson et al. 2009).

Despite the widespread mortality in forest stands, not all trees are affected, and the reasons for variations in host susceptibility remain uncertain. Tree vigor, a measure of wood production/leaf area, is significantly inversely correlated with individual tree susceptibility (Larsson et al. 1983; Mitchell et al. 1983) and is influenced by stand density, pathogenic agents, and fluctuations in precipitation and temperature (Gibson et al. 2009). Ponderosa pine trees are most likely to be susceptible in dense stands (>5.6 m^2^ basal area/hectare) of adult tress (>80 years) with diameters of 20–30 cm (Gibson et al. 2009). Tree mortality may increase consistently up to diameters of approximately 23 cm, with mortality of larger trees being more random (McCambridge et al. 1982).

Here, we compare radial growth rates of co-occurring populations of mature healthy and MPB-infected ponderosa pine trees collected at two sites in western Montana. Our objectives were to: (1) compare basal area increment values within populations and between sites; (2) examine the potential influence of changes in intrinsic water-use efficiency; (3) compare climate-growth responses; and (4) postulate on what increases individual tree susceptibility to MPB-induced mortality within a stand where other growth-influencing factors are held constant.

## Methods

### Tree-ring data collection

**Figure 1:**
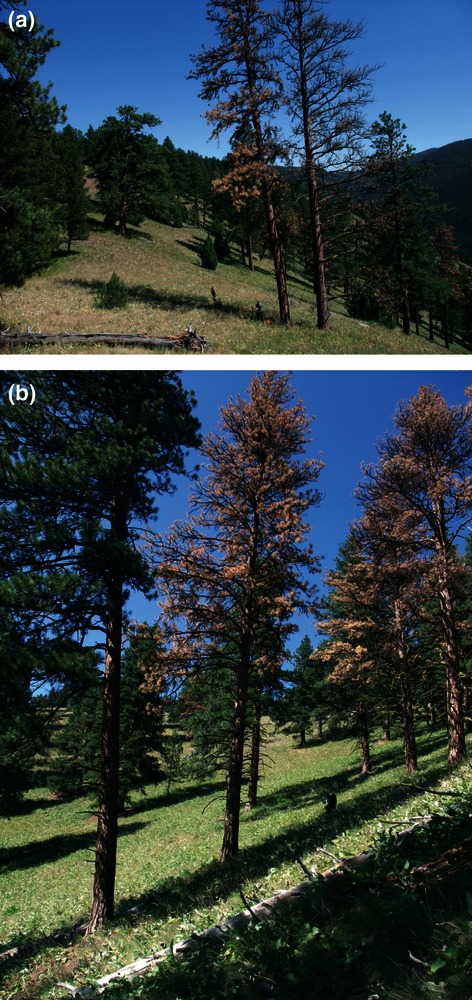
Visually healthy and MPB-infected trees at Kitchen Gulch, Montana. Photograph by authors, August 2011.

We collected samples in western Montana at Cabin Gulch Research Natural Area 28 km NE of Helena and Kitchen Gulch 32 km SE of Missoula (Table 1). The two sites were selected because they had a history of minimal anthropogenic impacts including livestock grazing, wood cutting, and fire suppression (pers. comm., Steve Shelly, USFS R1 RNA Coordinator) and contained both visually healthy and MPB-infected trees of multiple age classes growing in park-like stands (Fig. 1). At each site, we sampled visually healthy and MPB-infected trees using standard dendroecological field techniques, selecting older trees (>150 years) at each site using visual old-age characteristics. Only open-grown trees (Fig. 1) were cored to ensure that radial growth was not affected by canopy closure during, at least, the past century. Both sites are located on steep (25–40%), south-facing slopes with thin soils ranging from 20 to 50 cm (authors' observations).

**Table 1:** Site characteristics for Cabin Gulch and Kitchen Gulch

Site Name	Latitude (°N)	Longitude (°W)	Elevation (m)	Climate division	DBH- visually healthy (cm)	DBH-infected (cm)
Cabin Gulch	47.79	111.77	1140	Montana 4	32.44	27.53
Kitchen Gulch	46.71	113.65	1444	Montana 1	34.4	35.95

We identified visually healthy trees by the presence of green needles and no evidence of pitch tubes, boring dust, and live MPB eggs, pupae, larvae, or adults. MPB-infected trees were selected by the presence of red foliage in the crown and upon coring, blue-stained sapwood caused by the blue stain fungi. We additionally examined beetle gallery patterns on selected trees to confirm MPB activity opposed to other boring beetles. For each tree, we collected two core samples using increment borers at approximately 1.4-m height. Additionally, we measured crown height and basal diameter for each tree.

We processed collected cores using standard dendrochronology procedures (Stokes and Smiley 1968). We cross-dated the cores using the list method (Yamaguchi 1991), and confirmed dating accuracy using the diagnostic program COFECHA (Holmes 1983). We used raw ring-width data to calculate basal area increment (BAI, cm^2^ yr^−1^) for all cores from 1860 to 2007/8 for each of the four chronologies (CGR [visually healthy and developed from 12 trees and 20 cores] and CGI [MPB-infected; 10 trees, 18 cores] for Cabin Gulch and KGR (21 trees, 39 cores), and KGI (16 trees, 30 cores) for Kitchen Gulch). To account for the potential influences of variations in tree size and age between chronologies from the same site, we correlated unstandardized BAI values with standardized BAI values for each chronology. We found negligible differences between the chronologies (all *r*-values > 0.97) and then retained unstandardized BAI values for our remaining analyses as this allowed for direct comparisons of BAI values within and between our study sites.

### Carbon isotope analysis

We developed carbon isotope chronologies from both the visually healthy and MPB-infected trees at Cabin Gulch following methods discussed in Knapp and Soulé (2011). For each chronology, we selected six trees based on the longest and clearest ring structure, with two samples representing each tree. We manually separated tree rings for each sample into pentads (5-year segments) beginning in AD 1800 or later (because of lack of older cores) using a scalpel. We pooled the pentads from all samples and ground together to 40 mesh. To quantitate inter-tree isotopic variability, we also pooled samples for each 50-year period. We processed all ground samples to α-cellulose following conversion to holocellulose using the Jayme-Wise method (see Leavitt and Danzer 1993) and using batch processing and commercial digestion pouches (ANKOM Technology, Boston, MA). Sample extractions were first removed using a soxhlet extraction apparatus operating with toluene/ethanol and ethanol organic solvents followed by boiling in deionized (DI) water. Lignin was removed by reaction in an acetic acid-acidified, sodium chlorite aqueous solution at 70°C and then rinsed thoroughly in DI water. Final isolation of α-cellulose was achieved by treatment in 17% NaOH solution following methodology identified in Sternberg (1989) with samples being rinsed in DI water and dried at 70°C.

The α-cellulose was combusted to CO_2_ in a Thermo-Finnigan TC/EA and then delivered to a Thermo Finnigan Delta Plus XL mass spectrometer (Thermo Fisher Scientific Inc., Waltham, MA) operating in a flow-through mode with a Conflo III interface. We determined isotopic composition with respect to the PDB standard (Coplen 1996). For every four to five samples, a homogenous acetanilide working standard of known isotopic composition was run for mass spectrometer calibration to insure that machine precision of ca. 0.02–0.11‰ (SD) was present for each batch of 30–40 samples analyzed at the University of Arizona, Laboratory of Tree-Ring Research. Additionally, we used a holocellulose laboratory standard (MAWS) for approximately every 15 samples, which established precision of 0.12‰.

We determined intrinsic water-use efficiency (iWUE) following the equation of Ehleringer and Cerling (1995):


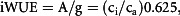
(1)

where A = CO_2_ assimilation of ponderosa pine leaves, g = leaf stomatal conductance, 0.625 = constant, and c_i_/c_a_ = intercellular CO_2_ and atmospheric CO_2_. We interpolated annual c_a_ with decadal observations beginning in 1800 (Etheridge et al. 1998) and with measurements collected at Mauna Loa from 1959 to 2009 (Keeling et al. 2008). Equations 2 (carbon isotopic discrimination for plants Δ) and 3 (the c_i_/c_a_ ratio; Farquhar et al. 1982) allowed us to obtain (c_i_):


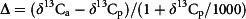
(2)


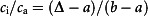
(3)

We derived the stable carbon isotopic composition of the atmosphere (δ^13^C_a_) from 1796 to 2002 using data from Francey et al. (1999) and Allison et al. (2003). Because yearly data were temporally incomplete until 1991, we used a sixth-order polynomial to create annual resolution data (e.g., Hemming et al. 1998). Holding all pre-1850 data stable at -6.4‰, we averaged δ^13^C_a_ values by pentad. We also averaged isotopic composition of the trees that were pooled by site (δ^13^C_p_) by pentad. Respectively, *a* and *b* represented constants for the discrimination during diffusion of CO_2_ into the air (4.4‰) and discrimination during carboxylation (assumed at 27‰).

## Statistical analysis

We used Spearman correlation (PASW Statistics 18.0) to identify the principal climatic drivers of radial growth for each site paired with data from climatic divisions (NCDC 1994) Montana-1 (MT1, Kitchen Gulch) and Montana-4 (MT4, Cabin Gulch) during 1895–2007/8. We examined monthly and seasonal precipitation, temperature, and drought severity influences, including lagged influences up to 1 year.

We examined potential possibilities for increased MPB susceptibility by comparing BAI values between visually healthy and MPB-infected populations at each site during stress and non-stress years using the non-parametric Mann–Whitney *U*-test. During 1895–2007/8, we categorized all years with Palmer Drought Severity Index (PDSI) values <−2.0 (moderate drought or greater) as stressful, or PDSI values > −0.5 (normal to wet) as non-stressful. We identified potential shifts in iWUE at Cabin Gulch following the methodology of Rodionov (2004) and Rodionov and Overland (2005). We used a significance level of *P* < 0.05 and cut-off length of 30 years to detect possible multi-decadal-length changes in iWUE since 1860.

## Results

### Climate/growth relationships

Climate–growth relationships indicated that the principal climate driver of growth was significantly correlated (*P* < 0.05) with July PDSI for each chronology. At Cabin Gulch, there was a large difference between chronologies (*r*_*s*_ = 0.42 CGR, *n* = 20 cores and *r*_*s*_ = 0.62 CGI, *n* = 18), but less difference existed between chronologies at Kitchen Gulch (*r*_*s*_ = 0.55 KGR, *n* = 39; *r*_*s*_ = 0.53 KGI, *n* = 30). During 1895–2008, July PDSI for MT4 had a downward linear trend (*r*_*s*_ = −0.34, *P* < 0.01) while July PDSI for MT1 exhibited no significant change (*r*_*s*_ = −0.11 *P* = 0.24), indicating increasingly drier summer conditions at Cabin Gulch and stable conditions at Kitchen Gulch. During the last decade (1999–2008), however, there was severe summer dryness at Cabin Gulch, with all years having negative PDSI values and a mean PDSI value of −2.61. Kitchen Gulch also experienced dry July PDSI conditions over the same period 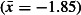, but 2 years had PSDI values > 0.

### Basal area increment

**Figure 2:**
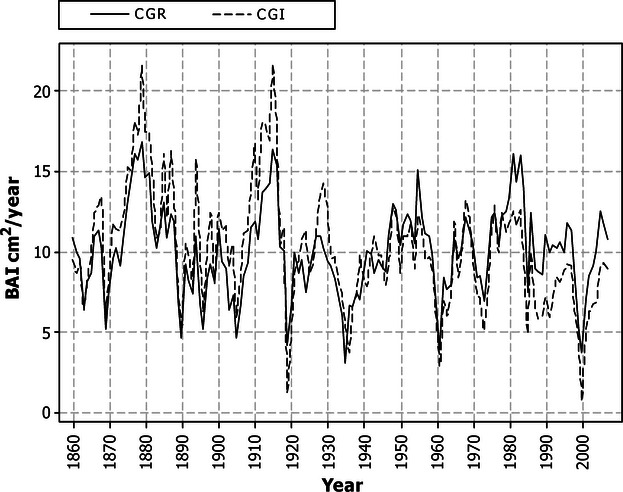
BAI values (cm^2^ yr^−1^) for the two chronologies developed at Cabin Gulch during 1860–2007.

**Figure 3:**
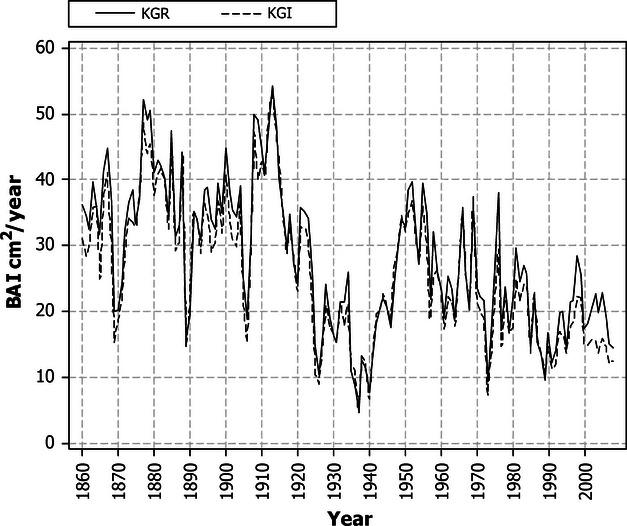
BAI values (cm^2^ yr^−1^) for the two chronologies developed at Kitchen Gulch during 1860–2008.

Basal area increment for MPB-infected and visually healthy trees at each site closely matched during the majority of the 150-year period (Figs. 2, 3), but diverged during the last two–three decades. Examining differences using 30-year intervals, mean BAI for visually healthy trees at Cabin Gulch was significantly less during 1890–1919 and significantly greater during 1980–2007 when compared with the MPB-infected sample (Table 2). At Kitchen Gulch, mean BAI was significantly greater during 1980–2008 when compared with the MPB-infected sample (Table 2) and for the average of two visually healthy chronologies compared with the average of the MPB-infected samples. The inflection point for radial growth divergence occurred ca. 1980 for Cabin Gulch, with the visually healthy trees maintaining higher BAI values for every year and the largest differences beginning in the mid-1990s (Fig. 2). A similar but less pronounced pattern occurred at Kitchen Gulch, with consistently higher BAI values for the visually healthy trees beginning in the early 1990s and the largest differences occurring during the last decade (Fig. 3). The combined chronologies showed no differences between infected and healthy trees until 1980–2007.

**Table 2:** BAI comparison between chronologies for MPB-infected and visually healthy trees at both sites and the two sites combined for 30-year periods (AD 1860–2007/8). Shaded boxes indicate significance at *p* < 0.05

Period (AD)	Chronology	Segment length (Years)	Mean BAI	Std. deviation (BAI)	Independent samples M–W U-test (one-tailed)
1860–1889	KGR	30	36.60	8.77	
	KGI	30	33.76	8.80	0.092
1890–1919	KGR	30	36.84	8.44	
	KGI	30	34.81	8.59	0.145
1920–1949	KGR	30	19.83	8.41	
	KGI	30	19.06	7.92	0.351
1950–1979	KGR	30	26.33	7.90	
	KGI	30	24.42	7.63	0.159
1980–2008	KGR	29	19.37	5.09	
	KGI	29	16.62	4.12	0.025
1860–1889	CGR	30	11.08	2.89	
	CGI	30	12.51	3.81	0.07
1890–1919	CGR	30	9.43	3.18	
	CGI	30	11.59	4.47	0.02
1920–1949	CGR	30	8.86	2.03	
	CGI	30	9.36	2.50	0.21
1950–1979	CGR	30	10.24	2.29	
	CGI	30	9.37	2.59	0.12
1980–2007	CGR	28	10.45	2.87	
	CGI	28	7.82	2.67	0.001

	Sites combined				
1860–1889	Visually healthy	30	23.82	5.55	
	Infected	30	23.14	5.89	0.282
1890–1919	Visually healthy	30	23.13	5.42	
	Infected	30	23.20	6.14	0.477
1920–1949	Visually healthy	30	14.34	4.71	
	Infected	30	14.21	4.59	0.474
1950–1979	Visually healthy	30	18.28	4.69	
	Infected	30	16.90	4.63	0.131
1980–2007	Visually healthy	28	15.06	3.46	
	Infected	28	12.29	2.97	0.002

Mean BAI values at Cabin Gulch were 18% higher (*n* = 27 years; *P* < 0.01, one-tailed) for the visually healthy trees compared with MPB-infected trees during stressful years (PDSI<−2.0), but the two populations were not different (*n* = 66; *P* = 0.781, two-tailed) during non-drought years (PDSI > −0.5). At Kitchen Gulch, BAI values of healthy trees were not significantly greater during either drought (*n* = 26 years, *P* = 0.19, one-tailed) or non-drought years (*n* = 69; *P* = 0.17, two-tailed).

### Intrinsic water-use efficiency

**Figure 4:**
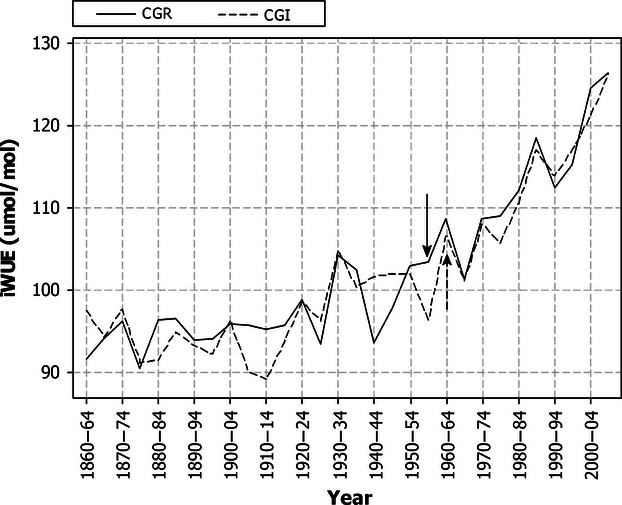
Intrinsic water-use efficiency (μmol/mol) for visually healthy (CGR) and MPB-infected trees (CGI) at Cabin Gulch (pentads from AD 1860–2009). Vertical arrows indicate regime shift (*p* < 0.05) inflection points.

Intrinsic water-use efficiency at Cabin Gulch was not significantly different (*P* > 0.05) for visually healthy and infected samples during 1860–2009 (101.5 *μ*mol/mol vs. 100.9 *μ*mol/mol) and 1980–2009 (118.3 vs. 117.7). Both samples experienced upward trends in iWUE, with significant (*P* < 0.05, 30-year cut-off) shifts toward higher iWUE beginning in 1955–59 for the visually healthy trees and 1960–1964 for the infected trees (Fig. 4). Mean iWUE between the early and shift points was 96.3 vs. 113.75 (18% increase) and 96.2 vs. 110 (17% increase) for the visually healthy and the MPB-infected populations.

## Discussion

### Radial growth divergence

Basal area increment values within populations and between sites were similar until the late 20^th^ Century, at which point the MPB-infected populations had consistently lower BAI values. Thus, growth rates 20–30 years prior to the current outbreak diverged between our selected populations with the slower growing trees being more vulnerable to infestation. These findings are consistent with the vigor hypothesis (e.g., Larsson et al. 1983), and the findings may be revealing in that these were open-grown trees sampled from the same slope, soils, and aspect as visually healthy trees. Sampled trees from the chronologies were often (approximately 50% of total) collected within 15 m of each other, further reducing the likelihood that growth differences were an artifact of microsite variability.

Although changes to higher iWUE occurred slightly earlier for the visually healthy chronology, iWUE trended upward for both chronologies at Cabin Gulch with only minor and non-significant differences between populations. Thus, changes in iWUE do not explain the growth divergences. Growth/climate responses also were similar, with both populations at both sites dependent on summer soil-moisture conditions as inferred from July PDSI values. At Cabin Gulch, the infected population exhibited greater growth sensitivity to climate, which is consistent with the findings of McDowell et al. 2010, who found greater drought-induced mortality in ponderosa pine with increasing climatic sensitivity. Conversely, at Kitchen Gulch, the healthy population exhibited slightly greater sensitivity to climatic variations than the infected population, suggesting that the relationship between vigor and climatic sensitivity may be site specific.

### Intrinsic water-use efficiency

Our findings also suggest that significant increases in iWUE do not confer immunity to MPB infestations for all trees during moisture-stress periods, but may have the potential to lessen the severity of the attacks because of the ameliorative effects of improved plant–water relationships. In the Northern Rockies, USA, radial growth rates and iWUE of ponderosa pine are significantly and positively associated with age, suggesting that the water stress-ameliorating effects of increased atmospheric CO_2_ preferentially favor older trees (Knapp and Soulé 2011). In this study, median tree age at CGR (260 years) and KGR (180 years) were greater than for CGI (207 years) and KGI (156 years). Our findings are consistent in that older trees may have been less affected by drought conditions than younger trees during the last 30 years, and in turn have been less susceptible to MPB infestations due to the reduction in moisture stress, particularly at Cabin Gulch where age differences (53 years) were more pronounced.

### Implications

The divergence in growth shown between populations at these sites may reveal “previously invisible genetic quirks*”* (Neese and Williams 1994:144) that may favor/disfavor different genotypes embedded within a local population when stress occurs. For both sites sampled, MPB-infected and visually healthy populations followed two different growth trajectories in the late 20th Century/early 21st Century, suggesting that some type of environmental shift favored one subgroup of ponderosa pine over the other prior to the onset of MPB attacks. In turn, the less-vigorous trees were unable to repel MPB attacks, making them more vulnerable to the current epidemic. For the Cabin Gulch and Kitchen Gulch chronologies, the visually healthy trees were less affected by drought conditions as they maintained higher BAI values during the past 20–30 years, although this relationship was better expressed at Cabin Gulch where the overall climatic conditions are drier than at Kitchen Gulch and age differences between groups were less. Conversely, the lack of pronounced superior growth of the healthy trees throughout the record suggests other possibilities for divergence. For example, prior MPB infestations that weakened, but did not kill, the trees 20–30 years ago may have made the MPB trees more susceptible to the current episode.

Overall these results were similar between the Cabin Gulch and Kitchen Gulch sites. Radial growth divergence occurred several decades prior to infection, suggesting that the likelihood of MPB infestation of individual trees older than 150 years is not arbitrary and potentially set in motion decades prior to actual infestations. In short, susceptibility of ponderosa pine to MPB infestations may be strongly associated with the long-term vigor of individual trees that emerges during episodic outbreaks. The consequences of these outbreaks are that they selectively remove the least vigorous trees from the local forest population, thus promoting an overall increase in tree vigor and drought resistance.

## Biographies

Paul Knapp is a professor and director of the Carolina Tree-Ring Science Laboratory in the Department of Geography at the University of North Carolina-Greensboro. His interests have focused on the paleoecological dynamics of western North American forests.

Peter Soule' is a professor in the Department of Geography and Planning at Appalachian State University. His main area of research deals with the aspects of climate and vegetation change in western North America.

Justin Maxwell is an assistant professor in the Department of Geography at Indiana University. His interest areas include climatology, dendrochronology, and forest disturbance in the southeastern and western U.S.
